# Ablation in Externally Applied Electric and Magnetic Fields

**DOI:** 10.3390/nano10020182

**Published:** 2020-01-21

**Authors:** Jovan Maksimovic, Soon-Hock Ng, Tomas Katkus, Nguyen Hoai An Le, James W.M. Chon, Bruce C.C. Cowie, Tao Yang, Yves Bellouard, Saulius Juodkazis

**Affiliations:** 1 Center for Micro-Photonics, Swinburne University of Technology, John Street, Hawthorn, VIC 3122, Australia; soonhockng@swin.edu.au (S.-H.N.); tkatkus@swin.edu.au (T.K.); ale@swin.edu.au (N.H.A.L.);; 2 ARC Training Centre in Surface Engineering for Advanced Materials (SEAM), School of Science, Swinburne University of Technology, John Street, Hawthorn, VIC 3122, Australia; 3 Australian Synchrotron, 800 Blackburn Road, Clayton, VIC 3168, Australia; brucec@ansto.gov.au; 4 Galatea Lab, IMT, STI, Ecole Polytechnique Fédérale de Lausanne (EPFL), Rue de la Maladière 71b, CH-2002 Neuchâtel, Switzerland; tao.yang@alumni.epfl.ch; 5 Tokyo Tech World Research Hub Initiative (WRHI), School of Materials and Chemical Technology, Tokyo Institute of Technology, 2-12-1, Ookayama, Meguro-ku, Tokyo 152-8550, Japan

**Keywords:** ablation, electric field, magnetic field, debris, femtosecond laser fabrication, silicon, near-edge X-ray absorption fine structure (NEXAFS)

## Abstract

To harness light-matter interactions at the nano-/micro-scale, better tools for control must be developed. Here, it is shown that by applying an external electric and/or magnetic field, ablation of Si and glass under ultra-short (sub-1 ps) laser pulse irradiation can be controlled via the Lorentz force F=eE+e[v×B], where v is velocity of charge *e*, E is the applied electrical bias and B is the magnetic flux density. The external electric E-field was applied during laser ablation using suspended micro-electrodes above a glass substrate with an air gap for the incident laser beam. The counter-facing Al-electrodes on Si surface were used to study debris formation patterns on Si. Debris was deposited preferentially towards the negative electrode in the case of glass and Si ablation. Also, an external magnetic field was applied during laser ablation of Si in different geometries and is shown to affect ripple formation. Chemical analysis of ablated areas with and without a magnetic field showed strong chemical differences, revealed by synchrotron near-edge X-ray absorption fine structure (NEXAFS) measurements. Harnessing the vectorial nature of the Lorentz force widens application potential of surface modifications and debris formation in external E-/B-fields, with potential applications in mass and charge spectroscopes.

## 1. Introduction

Ablation using ultra-short sub-1 ps laser pulses has become a popular method for three-dimensional (3D) material structuring: cutting, dicing, hole-drilling, surface- and volume-patterning with nanogratings [1,2,3,4], optical waveguide inscription in glasses and crystals [5], non-erasable optical memory and photonic crystals [6], creation of new materials and their high pressure and temperature phases by 3D confined micro-explosions [7], thermal morphing of laser fabricated 3D structures [8], laser-assisted etching [9], and light-induced back-side wet-etching [10]. Applications of colloidal nanoparticle synthesis by ablation in liquids [11] and laser-machining have become industrial applications with high throughput [12,13,14,15]. Burst-mode of fs-lasers and use of polygon scanners at surface beam travel speeds in excess of 100 m/s opens a new chapter for high average power ∼100 W high-repetition rate (with bursts) laser-machining.

In all the aforementioned studies, morphological changes on the surface or inside the volume of the irradiated sample are exploited for different applications, e.g., formation of nanogratings for optical retardance, wettability change of the surface, alteration of wet etchability of material, change in the debris formation during ablation, change of the ablation rate, etc. The possibility of influencing light-matter interactions inside the laser-affected focal volume, via external electric and magnetic fields, is a promising line of research for understanding fundamental aspects of laser-induced breakdown and solid state plasma.

For high precision fabrication, focusing optics with numerical aperture NA≈0.5 are used with focal spot 1.22λ/NA of (1–2) μm in diameter at visible wavelengths λ = (0.4–0.8) μm. Ablation using ultra-short sub-1 ps pulses at high irradiance 5–10 TW/cm${}^{2}$ begins with removal of electrons from the surface with heavier ions following after the negative charge [16]. This dynamic removal of material takes place in electrostatically coupled electron-ion systems. Hence, a valid question is whether an externally applied E-field (voltage *V* over a gap of Δd) or externally applied B-field can influence the dynamics of the ablation plume with asymmetry during the initial stage of negative-positive charge removal from the surface. This is of a particular interest since the ablation plasma is overall charge neutral.

Kinetic debris formation during ablation is a complex process, occurring in a dynamic ionized plasma where electron gain (reduction) and removal (oxidation) takes place during the flight of ionized debris. Debris at the laser treated regions are the result of interaction and neutralization of charged particles as well as their chemical modification in the ablation plume. Charged particles, electrons, and ions at high thermal speeds can be controlled and analyzed by application of electric and magnetic fields. Removal of ablation debris requires additional post-exposure steps and the smallest nanoparticles (tens-of-nm in diameter) usually cannot be removed without modification of the substrate. It is, therefore, essential to investigate possible control methods for both debris formation and deposition within the context of practical applications of laser processing. Nanoparticles of high refractive index materials are also promising for optical sensing applications [17]. Hence, sorting by size, shape and composition is required.

Here, a novel method for control over the ablation process is introduced using externally applied magnetic and electric B-/E-fields. Ablation of Si samples in different electric and magnetic field geometries show dependence via a displacement of debris patterns. Stemming from a recent demonstration of weak 0.1 T B-field influence on ablation by single fs-laser pulses [18], where it is shown that the B-field orientation affects the surface morphology of ablation ripples on Si, inciting an application for machining and sensing applications. Chemical analysis of ablated areas with and without a magnetic field showed strong chemical differences, revealed by synchrotron near-edge X-ray absorption fine structure (NEXAFS) measurements at the Si1s and O1s bands (method also known as: X-ray absorption near-edge structure (XANES)). NEXAFS probes energies up to ∼50 eV above the X-ray absorption edge of a particular core shell of an element and the spectra produced are sensitive to the chemical bonding associated with that element. The X-ray fluorescence mode was used during NEXAFS detection which probes a depth comparable to the X-ray transmission depth. Adsorbates even at a sub-monolayer coverage can be detected and, due to the dipole selection rules of X-ray absorption, with suitable samples the molecular orientation can be determined using several discrete measurements with polarized X-ray radiation [19].

## 2. Experimental: Samples, Fabrication, Characterization

A femtosecond (fs-)fiber laser (Yuzu, Amplitude Ltd., Pessac, France) operating at λ=1030 nm, $t_{p}=270$ fs pulse duration and up to 2 MHz repetition rate was used for glass ablation. Direct fs-laser writing by ablation of Si was carried out with a solid state Yb:KGW laser (Pharos, Light Conversion, Ltd., Vilnius, Lithuania)at the same wavelength with $t_{p}=230$ fs pulses and repetition rate up to 0.6 MHz. High precision mechanical stages (Aerotech, GmbH, Pittsburgh, PA, USA) were used for in-plane (xy) translation of the sample. A software-hardware integration solution of the entire fs-fabrication unit was made by Altechna. Ltd., Ablation was carried out at different focusing and scanning conditions but always at a strong pulse-to-pulse overlap (more than 90%) at repetition rate of 200 kHz or specified where different. Laser ablation of Si was carried out in air (cleanroom, class 1000 or ISO 6). Crystalline c-Si (n-type; Phosphorus-doped) 〈100〉 and intrinsic samples (University Wafer, Boston, MA, USA) were used with presence of E- and B-fields, and ablation was carried out with the Pharos laser system. All samples were cleaned in acetone and isopropyl alcohol (IPA) prior to use to minimize surface contamination. Ablation conditions varied from single to heavy overlapping pulses, of low and high energy (50–500) nJ, with objective lenses of numerical aperture NA=0.14, 0.4 and 0.7 (Mitutoyo, Kawasaki, Japan); indicated where applicable. Pulse energy given in the text was recalculated for the sample’s surface.

The strength of an external electric field applied during laser ablation was changed up to 2 V/ μm (air breakdown at normal conditions is at 3 V/μm) using a 650 V/50 mA power supply unit (TDK-Lambda Z650-0.32-U, Mouser Electronics, Mansfield, TX, USA). Electric field electrodes used during laser ablation were produced with photolithography using laser fabricated masks. A silica layer was sputtered over and between the Al-electrodes to avoid discharge when applying strong electric fields (as an extra precaution) through the laser breakdown region at the air-Si interface. In case of soda-lime glass ablation, the electrodes were made by a simple assembly of Au-coated V-shaped electrodes, fabricated by a femtosecond laser according the process described ref. [20].

An external magnetic B-field (N→S) was applied using Nd${}_{2}$Fe${}_{14}$B magnets of different size and geometry. B-field was applied perpendicular to the surface of Si samples; orientation was changing at the edge regions. The B-field strength was directly measured at the ablation surface and ranged from B = 0.02–0.16 T. A Gauss probe with 2-axis Magnetic Field sensor (Xplorer GLX PS-2002, Pasco, Roseville, CA, USA) was used. Magnetic washers with a central hole were implemented for ripple pattern ablation and debris harvesting on cover glass (facing the ablation region).

NEXAFS was carried out to identify chemical modification of laser ablated regions on Si at Si1s and O1s spectral bands at the high throughput inline station of Soft X-ray beamline of the Australian Synchrotron, ANSTO. It operates at high vacuum $10^{-7}$ mbar pressure at room temperature (RT). The total fluoresence yield (TFY) mode was used. Polarization was elliptical with the beam size of 1×1 mm${}^{2}$ and resolution Δ E/E = (5–10) ×$10^{3}$. Flux at 400 keV was ∼1 ×$10^{12}$ Photons/s/200 mA at the sample.

Structural and optical characterization of laser ablated regions and debris fields was carried out by dark-field microscopy (ECLIPSE Ti-S microscope, Nikon, Tokyo, Japan) and scanning electron microscopy (Raith EBL 150${}^{\mathrm{TWO}}$, Raith, Dortmund, Germany), respectively.

## 3. Results

### 3.1. Ablation of Glass in External E-Field

**Figure 1:**
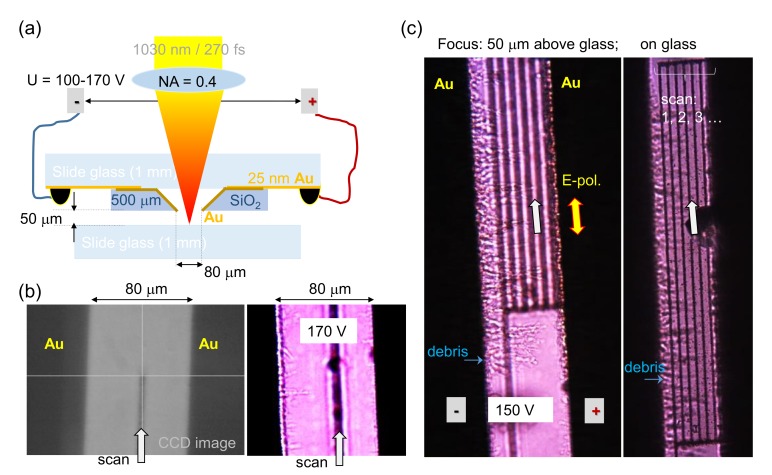
(**a**) Schematics of suspended micro-electrodes above glass surface subjected to ablation. (**b**) An in situ video camera image during scan of the laser beam (**left**) and debris observation at the edge of the electrode (**right**). (**c**) Optical images after laser ablation with focal plane at the electrodes’ edge and on the surface of the sample. Ablation was carried out at irradiance of ∼5 thresholds (the threshold fluence $F_{p}=2$ J/cm${}^{2}$) of glass ablation by single fs-laser pulses at scanning speed $v_{s}=10\;\mu$m/s.

Figure 1a shows the geometry of the experiment where the opening of a slit was aligned with the linear movement along y-axis translation of integrated stage. The ablation threshold of glass 2 J/cm${}^{2}$/pulse [21] was exceeded by approximately 4–5 times at a repetition rate of 750 kHz. Electrical DC field of up to 2 V/μm was applied during laser ablation. A fixed 50 μm separation between the glass and the electrode tip was maintained during laser scanning. Several lines were ablated with their different lateral position within the slit (Figure 1b,c; scans progressed from negative towards positive electrode in (c)). Polarization was made parallel to the scan direction ($E_{l}\|v_{s}$) to avoid force acting towards the edge of the slit contact $\left(e\cdot E_{l}\right)$; $E_{l}$ is the light field. Accumulation of debris on the negative electrode was observed as revealed by optical imaging (Figure 1b,c). A linearly elongated debris pattern protruding from the electrode is consistent with deposition of charged debris at the tip of formation where the strength of the electrical field and its gradient is the highest. It is noteworthy that the as-formed linear patterns were stable under switching voltage on and off (for the same polarity).

It was observed that the polarization of laser pulses which defines the orientation of oscillating electrons in the applied light field was not an important factor for formation of the linear debris structures on electrodes observed experimentally. A linearly ordered agglomeration of glass debris micro-particles were protruding from the negatively biased electrode as shown in Figure 1c.

### 3.2. Ablation of Si in External E-Field

**Figure 2:**
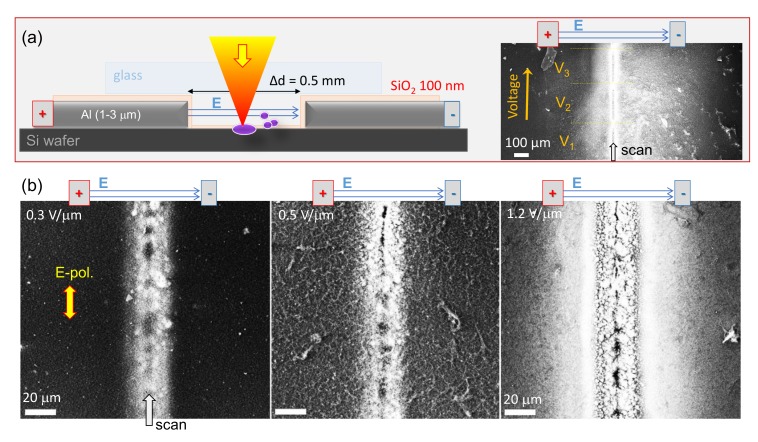
(**a**) Schematics of the experiment: ablation of intrinsic c-Si with Al-electrodes deposited onto sample. Silica layer of 100 nm was sputtered to prevent stochastic discharge between electrodes. SEM image of three regions ablated at different applied voltages $V_{3}>V_{2}>V_{1}$. Debris pattern was shifted towards the negative electrode. (**b**) Debris field distribution at different E-field strengths: 0.3, 0.5, 1.2 V/μm, respectively. Irradiation conditions: pulse energy $E_{p}=150$ nJ (fluence $F_{p}=0.24$ J/cm${}^{2}$/pulse), scanning speed $v_{s}=1\;$mm/s, repetition rate f=200 kHz, focusing through a 1.1 mm thick borosilicate glass with NA=0.14 objective lens.

The debris patterns for the Si ablation case was explored using SEM imaging. Crystalline un-doped and phosphorous-doped Si were used. To avoid a spontaneous electrical discharge between electrodes placed directly on Si or due to an air breakdownduring ablation, a 100-nm-thick SiO_2_ layer was deposited over the Al-electrodes (Figure 2a). Laser irradiation conditions remained constant, which was approximately more than double 0.24 J/cm${}^{2}$ (Figure 2) the ablation threshold 0.1 J/cm${}^{2}$/pulse of Si. Due to a strong overlap between adjacent pulses—$N=1.8\times 10^{3}$ pulses per diameter of 1.22λ/NA=9μm—ablation was strong despite low fluence per pulse.

Displacement of the Si debris field toward the negatively biased electrode was discernible with increasing applied E-field strength as shown in Figure 2a. Three segments on the right show ablated debris with increasing voltage *V* applied to the electrodes separated by Δd=500μm. It was apparent from SEM observation of surface charging (bright areas in SEM image) that larger debris fields were formed at larger applied voltages (E-field strengths). A lower SEM magnification afforded better contrast of the debris field, revealing the asymmetry which was not evident in the high magnification image, where the symmetric central groove was the dominant feature (Figure 2b). The central part has most of agglomerated material which charges strongest and dominates the contrast of SEM image taken by back-scattered electrons (Figure 2b). Samples were observed as ablated without surface cleaning. Polarization was kept parallel to the scan direction as in the case of glass ablation (Figure 1).

There was no qualitative difference in formation of ablation patterns with and without glass cover over the gap between Al-electrodes (Figure 2a). Tendency of the Si debris field deposition towards the negative electrode agreed with the above discussed glass ablation patterns (Figure 1).

### 3.3. Ablation of Si in External B-Field

**Figure 3:**
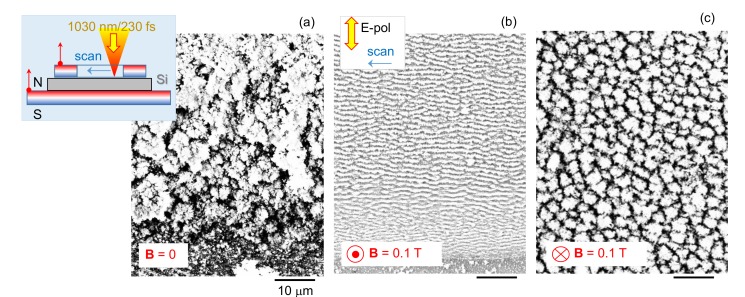
Ripple patterns ablated on c-Si (n-type) in the presence of different orientations of the magnetic field B≈0.1 T (directly measured for the used geometry). (**a**) SEM image (top-view) of the fs -ablated region without magnetic field. (inset) Schematic of magnetic field geometry during laser ablation. (**b**,**c**) Ablation patterns with two opposite directions of the magnetic field: out-off-surface irradiated by fs pulses (**b**) and along the propagation of laser beam (into the surface; (**c**) Conditions of raster scan: pulse energy $E_{p}=570$ nJ (fluence 22.5 J/cm${}^{2}$/pulse), scanning speed $v_{s}=1000\;\mu$m/s, spacing between raster lines Δ y = 2.5μm, laser repetition rate f=200 kHz, focusing with NA=0.7 (into 1.8 μm diameter spot ), polarization of incident light was perpendicular to the raster scanning direction (inset in (**b**)). Magnetic field orientation N→S is defined by the top-face of magnet (see red-arrow in the inset in (**a**)); top magnet is circular with a hole, bottom magnet is flat-continuous).

A surface morphology change was observed during ablation in an externally applied magnetic field for nanosecond [22] and femtosecond [18] single laser pulses. Strong asymmetry in the ablation debris patterns were observed when a Si sample was placed on a flat magnet (B parallel to the Si surface normal) for laser ablation [18]. Here, patterns were studied when formed by laser ablation under raster scan conditions with strongly overlapping laser pulses, while a Si wafer was sandwiched between two magnets; the top magnetic washer has a hole (see inset of Figure 3). The magnetic field was normal to the sample’s surface in this study. Ablation was carried out over extensive ∼1 mm${}^{2}$ areas at the conditions of ripple formation (overlapping pulses). SEM observation of laser patterned regions was carried out without surface cleaning to better reveal differences induced by orientation of B-field.

When Si samples were laser ablated while being sandwiched between a flat magnet and a magnetic washer, a strong difference of the ablated surfaces was obvious (Figure 3). With the B-field facing out of Si surface (Figure 3b), a typical ripple pattern with period Λ≈λ was observed and the orientation of ripples corresponds to the most usual case of wavevector of the ripple pattern parallel to the E-field $k_{\Lambda }\|E_{l}$. The bottom-right region of the image (Figure 3b) was slightly closer to the top washer-magnet, where some inhomogeneous B-field would be expected. This was also the end of scan sequence. These factors might contribute to unevenness in ripple period. For the opposite B-field orientation (Figure 3c), distinctly different patterns with a coarser micro-structure was observed. Such hillock-like morphology would be consistent with ablation pattern formed under circularly polarized irradiation; however, polarization was linear (same as in Figure 3b). In both cases of B-field orientation, the pattern was recorded in the central location of the magnetic washer hole where B-field was the most uniform (but weaker as compared with that at washer’s edge). Si ablated without magnets under same irradiation conditions showed a strongly oxidized micro-rough structure (Figure 3a). Beneath that surface, a ripple pattern similar to that in Figure 3b would emerge after short HF washing for removal of the oxide layer (see Section 4 for discussion). Laser ablation was carried out at very high 22.5 J/cm${}^{2}$/pulse fluence at strongly overlapping conditions with only 5 nm travel distance between adjacent laser pulses when focal spot was 1.8 μm. This is the main reason of very strong debris accumulation towards the end of ablation scan (bottom-right corner Figure 3a).

### 3.4. NEXAFS of Si Ablated in External B-Field

**Figure 4:**
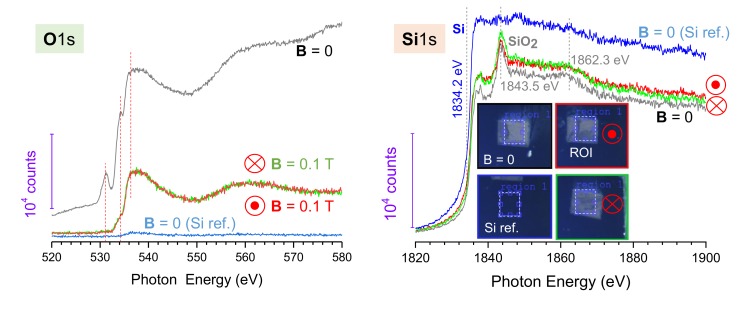
Synchotron NEXAFS spectra of O1s and Si1s bands from silicon ablated samples with and without externally applied magnetic fields. Pristine Si was used as a reference (inset in Si1s plot show optical images of the samples and region of interest (ROI) from where NEXAFS spectra were taken). Refer to Figure 3 for geometry and surface morphology imaging. Ablation conditions: pulse energy $E_{p}=570$ nJ, scanning speed $v_{s}=1$ mm/s, spacing between raster lines Δy=25μm, focusing with NA=0.7, inline pulse separation Δx=5μm, ablated area 1.25×1.25 mm${}^{2}$.

The near-edge X-ray absorption fine structure (NEXAFS) technique was used to identify chemical modification in the laser ablated regions. It can detect phase modifications of olivine induced by fs-laser ablation via change of spectral signatures at the Fe${}^{3+}$ and Fe${}^{2+}$ spectral bands [23]. NEXAFS uses methods based on electron yield detection (EYD) and is sensitive to the top 10 nm of the surface. NEXAFS recorded in the total fluorescence mode was recorded alongside EYD. These measurements have a sampling depth comparable to the attenuation length of the X-rays which will be in the range 0.4 to 0.8 μm at the O1s edge and 1.2 to 2.4 μm at the Si1s edge. The difference in attenuation length is calculated against that of a pure SiO_2_ and a pure Si matrix, respectively. It confirmed formation of an oxide layer on the surface in the laser ablated patterns without magnetic field B=0 T (Figure 4). NEXAFS measurements were made on the Soft X-ray of the Australian Synchrotron over the Oxygen and Silicon 1s absorption edges. The X-ray beam size used was unfocused and was 1×1 mm${}^{2}$, each measurement was checked to lie well within the total laser ablated regions which were of 1.25×1.25 mm${}^{2}$. NEXAFS intensity due to the surface oxide was considerably stronger compared to the native oxide layer (∼2 nm) of the reference Si sample (Figure 4). Si was ablated in the same configuration as the ripple patterning (Figure 3), only without the top magnet washer. Pristine Si was used as a reference sample for comparison of the O1s and Si1s signatures from native oxide (Figure 4). In the case of two opposite orientations of the B-field, there was no difference in the spectra at O1s and Si1s bands. NEXAFS intensity counts were proportional to the pulse energy used for ablation. Interestingly, the Si1s spectra showed lower intensity from the ablated regions as compared to those from the reference Si. This could be rationalized as a considerably thicker SiO_2_ layer in the ablated area overlying an undisturbed Si substrate. It is difficult to quantify the thickness of this overlayer from these spectra, but it must be tens to 100 nm thick. Clear signatures of amorphous (disordered) SiO_2_ are recognizable with characteristic X-ray peaks at 1843.5 eV and 1862.3 eV and is consistent with those of silicate spectra [24]. As expected, the edge of pure elemental Si absorption/emission is ∼9 eV lower in energy when compared to the SiO_2_ peak (Figure 4) [25]. A smaller amount of distinct spectral features is consistent with disordered orientation of chemical bonds probed in the NEXAFS measurement, which is proportional to ${\left|E\cdot O\right|}^{2}$ or $\cos^{2}\left(\delta \right)$, where O is the direction of final state orbit, E is the E-field vector of the incident beam E and δ is the angle between them [26] (see further discussion in Section 4).

### 3.5. Debris Harvesting from Si Ablated in External B-Field

**Figure 5:**
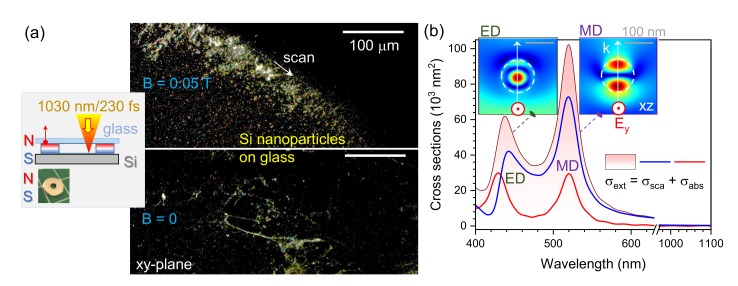
(**a**) Ablation of c-Si (n-Type) in magnetic field of B≈0.05 T with debris collection on a transparent cover glass collector substrate. Schematic of magnetic field geometry during laser ablation (top). Optical dark-field images of the collector substrates for ablation with and without magnetic field, respectively; non-magnetic washer of the same geometry was used for the B=0 T ablation. Irradiation conditions: pulse energy $E_{p}=100$ nJ, scanning speed $v_{s}=10$ mm/s, repetition rate f=200 kHz, focusing through a cover glass (collection substrate of 130–170 μm thickness) with a NA=0.14 objective lens. (**b**) Finite difference time domain (FDTD; Lumerical) modeling of extinction, absorption and scattering cross sections $\sigma_{ext}=\sigma_{abs}+\sigma_{scat}$ of a 120-nm-diameter Si nano-sphere in air. Insets show E-field maps at the electrical dipole ED 437 nm and magnetic dipole MD 520 nm peak wavelengths, respectively; the scale bars are 100nm, the arrow shows the light propagation direction and the intensity scales are (0–3.2) for ED and (0–2.8) for MD.

Figure 5a shows dark-field images of Si debris nano-/micro-particles collected on a cover glass which was placed over the central hole of a magnet washer. Laser beam was scanned approximately 0.1 mm from the edge of the magnet following a curved trajectory. The diameter of the focal spot was d∼9 μm and approximately N=180 pulses where overlapped during the dwell time $d/v_{s}$ (time to cross distance *d*). In the presence of B-field, the curved trajectory of the beam scan is recognizable in the pattern of deposited nano-/micro-particles. Propensity of debris deposition was observed in the direction towards the hole center. Less debris without a clear pattern edge were observed when ablation was carried out inside a non-magnetic washer at the same conditions. Thickness of the washers used was ∼0.5 mm. The range of colors in dark-field image corresponds to nanoparticles of different size and composition. Evidently, the larger lateral spread of ablation debris in the presence of a magnet is also related to the larger plume height. This would be expected and was revealed in this experiment (Figure 5 a).

Figure 5b shows the calculated values of absorption and scattering cross sections of a 120 nm diameter Si sphere. A finite difference time domain (FDTD, Lumerical) method was used for simulations with total-field scattered-field approach where the incident plane wave has E=1 strength. A strong field enhancement at the electrical and magnetic dipole spectral positions ED and MD, respectively, over the blue-green spectral span is present as shown in the cool-hot color map of the central cross section of a nano-sphere (insets in Figure 5b). Strong scattering resonances qualitatively explain the principle of color formation in the dark-field image of different size debris nanoparticles (Figure 5a). At the resonances, absorption and scattering cross sections exceed the geometrical one $1.1\times 10^{4}$ nm${}^{2}$ several times.

## 4. Discussion

Both external E- and B-fields caused larger lateral spread of debris fields. The drift of charged ablated particles (ions and electrons) in an externally applied E-field was dominated by the dynamics of heavier positive charges which moved preferentially towards the negative electrode. Apparently, the lighter electrons were compensating for the charge non-uniformity in the ablation plasma plume rather defining the directionality of the plume’s drift. This tendency was confirmed for glass and Si nano-/micro-particles ablated (formed) in the ablation plume with different electrode geometries.

The observed wider lateral distribution of debris as well as reaching larger heights (Figure 5) should be related to the separation of positive and negative charges in the presence of B-field. When the velocity of the ablation plume *v* is parallel to the magnetic field *B*, there is no lateral charge separation since the Lorentz force [v×B]=0. This corresponds to the initial stage of ablation when Si was placed on a magnet. The charges ±|q| experience a cyclotron spinning around the B-field lines with a frequency defined by their mass $\omega_{c}=qB/m$. The radius of the spinning trajectory is $r_{c}=mv/\left(qB\right)$. When the charges start to move at an angle to the magnetic field lines at later stages of the plume formation, lateral separation of electrons and ions existing in the laser ablated plume takes place. Such charge separation and acquisition of an angular momentum favors longer debris travel times and distances. In turn, this caused a longer oxidation time and SiO_2_ formation which comprises some of the debris on the surface of Si samples used. In SEM images (Figure 3), SiO_2_ formation is demonstrated by a strong surface charging, spreading over a larger surface area which can explain differences in X-ray fluorescence at the O1s band for B=0 and B≠0 cases (Figure 4); in the latter, debris are more spread causing lower fluorescence counts from the ROI. The strong influence of magnetic field orientation on ripple formation can be understood from geometry of ablation under tight focusing conditions, when the focal spot is ∼2 μm in diameter, and when ablation is carried out in a strongly overlapping pulse regime. This favors a strong charge separation laterally from the very early stages of ablation (electrons and ions are moving at angle to the B-field).

NEXAFS data invites further investigation of chemical modifications. Flatter and higher intensity NEXAFS spectra should be related to surface roughening which corresponds to an effective increase of orientation angles O. A wider range of angles smoothed the overall ${\left|E\cdot O\right|}^{2}$ product [26]. The bonding, i.e., transition direction becomes nearly parallel to E when the beam strikes the surface at a grazing angle (due to a non-flat surface). In the reference Si sample, the strongest signal was from the Si-band, with the weaker amorphous a-SiO_2_ feature observed at 1849.9 eV photon energy (Figure 4) due to multi-scattering transitions [27]. Also, a shift of spectral peaks towards larger energy is expected in denser silica phases [27] and is expected at the high irradiance conditions used [28]. The prominent 531.5 eV O1s band observed in Si ablated without magnetic field (Figure 4) is consistent with adventitious carbon C=O [29]. For further quantitative insights into surface chemical and structural modifications at specific irradiation conditions in the presence of B-field, NEXAFS data analysis will be carried by the readily developed program [30]. Since NEXAFS is sensitive to the bond orientation, it could be useful for the investigation of unusual magnetism of ablation debris as it was observed in carbon nano-foam made by laser ablation [31]. Debris formation in strong inhomogeneous E-/B-fields are expected to create new pathways for nanomaterial synthesis and sorting as discussed next.

Sorting and controlling of nanomaterials is another challenge which could be tackled using dielectrophoretic (DEP) force exerted on nanoparticles by strongly inhomogeneous electric field using wedge-contacts as illustrated in (Figure 1a). The DEP force is acting on an induced dipole d=xQ in a gradient of an applied electrical field $E_{a}\left(\omega_{a}\right)$ oscillating at the frequency $\omega_{a}$: $F_{DEP}\left(\omega_{a}\right)=d\cdot \nabla E_{a}\left(\omega_{a}\right)$, where *Q* is the induced charge and *x* is its separation (length of the dipole). The induced dipole depends on polarizability, which is material, size, and medium dependent and is expressed by the Clausius-Mossotti factor $K(\omega_{a})$; then $F_{DEP}\left(\omega_{a}\right)=K\left(\omega_{a}\right)\nabla E_{a}\left(\omega_{a}\right)$. It provides the possibility of sorting nanoparticles based on their resonance. For a sphere radius *r*, K=2πr3εmReεp*−εm*εp*+2εm*, where the complex permittivity (dielectric constant) of the particle and medium are $\varepsilon_{p,m}$, respectively, and the frequency dependence is governed by a resonance with $\varepsilon^{*}=\varepsilon +i\sigma /\omega_{a}$; σ is the electrical conductivity. The control of the DEP force for size, shape and material sorting will require the fabrication of micro-contacts with small separation, which is technically feasible. In this study, we used electrostatic E-field which should be frequency tuned to deliver DEP sorting.

## 5. Conclusions and Outlook

It was demonstrated that distribution of glass and Si ablation debris is affected by external B- and E-fields in terms of directionality of redeposition, amount of oxide formation on Si, and morphology of surface ripples.

It is promising to apply external B-/E-fields during fs-laser structuring inside polymers, solid state dielectrics and semiconductors where light-field polarization effects have been demonstrated to influence morphology of structured volume [32,33,34,35,36]. Engineering of permittivity via optically induced dielectric-to-metal (Die-Met) [37] transition with ultra-short laser pulses is another phenomenon which can be explored in the presence of externally applied fields to guide material relaxation. Investigating the impact of externally applied B-/E-fields on laser structuring by ultra-short Gauss-Bessel laser pulses [38,39], influence on surface ripple formation [40], nonlinear wavelength conversion [41] and photo-refractive properties [42] is expected to reveal new methods of modification control. The vectorial control of light-matter interactions via the Lorentz force of photo-excited carriers (electrons, holes, ions) is expected to enrich the available toolbox of material structuring. Use of a magnetic field at high light intensities discussed here could be adopted in currently active field of research in topological photonics [43,44] and applied for breaking an optical reciprocity and parity-time symmetry.
